# A mixed methods PAR study investigating social capital as a resource for Black and other racially minoritised communities in the UK: A study protocol

**DOI:** 10.1371/journal.pone.0296125

**Published:** 2023-12-21

**Authors:** Georgina Gnan, Zara Asif, Sanchika Campbell, Jacqui Dyer, Anna Ehsan, Katrin Hoffmann, Hanna Kienzler, Shabbir Mellick, Nathaniel Martin, Cheryl Osei, Abreen Rebello, Imade Remouche, Rebecca Rhead, Denise Richards, Ibrahim Sabra, Sara Sabra, Pippa Sterk, Charlotte Woodhead, Stephani Hatch

**Affiliations:** 1 Department of Psychological Medicine, Institute of Psychiatry, Psychology and Neuroscience, King’s College London, London, United Kingdom; 2 ESRC Centre for Society and Mental Health, King’s College London, London, United Kingdom; 3 Faculty of Social Science and Public Policy, Department of Global Health and Social Medicine, School of Global Affairs, King’s College London, London, United Kingdom; 4 Black Thrive Global, London, United Kingdom; 5 Centre for Global Mental Health and Health Services and Population Research Department, Institute of Psychiatry, Psychology and Neuroscience, King’s College London, London, United Kingdom; 6 Faculty of Social Science and Public Policy, School of Education, Communication and Society, King’s College London, London, United Kingdom; University of Alabama at Birmingham, UNITED STATES

## Abstract

Understanding how different Black and other racially minoritised communities thrive is an emerging priority area in mental health promotion. Literature demonstrates health benefits of social capital (social resources embedded within social networks). However, its effects are not always positive, particularly for certain subpopulations who are already disadvantaged.The CONtributions of social NEtworks to Community Thriving (CONNECT) study will use Participatory Action Research (PAR) to investigate social capital as a resource that benefits (or hinders) racially minoritised communities and their mental health. The CONNECT study was designed within a partnership with community organisations and responds to local policy in two South-East London Boroughs, thereby providing potential channels for the action component of PAR. Taking an anti-racism lens, we acknowledge the underpinning role of racism in creating health inequities. We apply an intersectional framework to be considerate of overlapping forms of oppression such as age, gender, socioeconomic status, and sexual orientation as an essential part of developing effective strategies to tackle health inequities. Key components of this mixed methods PAR study include (1) involving racialised minority community members as peer researchers in the team (2) collecting and analysing primary qualitative data via interviews, photovoice, and community mapping workshops, (3) developing relevant research questions guided by peer researchers and collaborating organisations and analysing secondary quantitative data accordingly, (4) integrating qualitative and quantitative phases, and (5) working closely with community and policy partners to act on our findings and use our research for social change.The PAR approach will allow us to engage community (voluntary sector and government) and academic partners in decision making and help address imbalances in power and resource allocation. Knowledge generated through this collaborative approach will contribute to existing community initiatives, policies, and council strategies. This will ensure the views and experiences of racially minoritised communities drive the changes we are collaboratively committed to achieving.

## Introduction

Understanding how different Black and other racially minoritised communities thrive is an emerging priority area in mental health promotion [1]. Moreover, we need to better understand how experiences of thriving are connected to and influenced by social networks and resources, and subsequent “social capital”. We use the term “racially minoritised”, to acknowledge that “minoritisation” is a social process shaped by power and systemic disadvantage, in which dominant groups have and continue to contribute to the structural oppression of Black and other racially minoritised groups [2,3]. We consider “thriving” to be a living concept, rather than a static or internal construct [4] and to mean realising one’s potential, living a happy and healthy life, having meaning and purpose, and having fulfilling relationships [5,6].

The CONtributions of social NEtworks to Community Thriving (CONNECT) study aims to learn about how Black and other racially minoritised communities thrive through improving understanding of “social capital” [7]; the nature and influence of social networks and the resources embedded within (such as social support, information channels, social credentials) [8] and how they contribute to social inequities and subsequent mental health. Understanding how social capital and networks operate around issues such as (1) community safety, (2) violence against women and girls, (3) skills and employment, and (4) food security, can teach us about what makes communities thrive, the resources they need, and how this can be collectively supported, nurtured, and sustained in the future. We recognise that people’s ability to “thrive” will depend to a significant extent on the structural, material, political, economic, social, environmental and ideological environments they inhabit [4,6]. Therefore, we put questions of structure, power, context, access to resources and injustice front and centre. We will use a Participatory Action Research (PAR) approach, which will allow for us to (1) investigate the benefits of social relationships in context, (2) help build trusting, sustainable relationships, which are likely to increase the novelty and impact of the research findings [3,9] and (3) support research focused on change and improvement rather than describing the status quo [10].

A large body of empirical work suggests that deep and meaningful close relationships play a vital role in promoting thriving [11–13]. This research shows that people who are more socially integrated and experience more supportive and rewarding relationships with others have better mental health, higher levels of subjective well-being, and lower rates of morbidity and mortality. Our social relationships also provide resources that may create economic, and other, opportunities through the generation and accrual of “capital”, for example including skills and credentials facilitated by one’s relationships [14]. Bourdieu framed social capital as accumulated actual or virtual resources acquired by individuals and maintained that social capital resides in the individual and is linked to social connections that a person can utilise for advancement [15]. Bourdieu’s work emphasises structural constraints and unequal access to institutional resources based on class, gender, and race.

Although there is a wealth of literature demonstrating the health benefits of social capital, it is also acknowledged to be a “double-edged” phenomenon, as its effects on health are not always positive [16]. This is particularly so in certain subpopulations [9], with some groups benefitting from the systemic exclusion of other groups, often based on race, class and geography [17]. For example, the same strong ties that bring benefits to members of one group (e.g., white people using social capital for economic advantage), can inhibit others, such as those from racially minoritised backgrounds, from accessing it [18]. This results in the inequitable pattern of health outcomes through limited connections with people in power, fostering group intolerance, excluding some groups from resources, or even causing division and strife within minoritised communities over scarce resources [17]. Furthermore, racism and discrimination profoundly shape people’s environments and opportunities, affecting healthcare access and experiences, formal education, informal networks, jobs, and careers, increasing the likelihood of facing poor quality housing, neighbourhood deprivation and violence, and food insecurity [19–22]. Social capital has been related to racial disparities in health at the individual and at the group level [23]. Therefore, social capital needs to be taken seriously as a determinant of health disparities [17] and there is a need for improved understanding about how such social networks and resources, and subsequent social capital, may play a role in different Black and racially minoritised communities. Research has emphasised the need to look at population subgroups, with an intersectional focus, to better disentangle the relationship between social capital and health [9].

There is little consensus on how to build or strengthen social capital [24]. However, Brune and Bossert [25] describe general principles on how to foster social capital, such as building on existing organisation in communities and developing participation mechanisms (e.g., developing management and leadership skills of community members with the goal of strengthening community organisation and self-management). The simultaneously positive and negative roles that social networks play within and between systematically disadvantaged groups, and how these are embedded in communities, can be harnessed using co-production approaches (e.g., PAR). Previous research has also highlighted the importance of community-based participatory research approaches for authentic voices in fostering and sustaining transformational change in policies and practices that are driving the social determinants of health and wellbeing [26]. These approaches aspire to benefit and centre the voices of marginalised groups [27].

### Project development

This study builds on over a decade of our research and engagement work that has focused on identifying ways to reduce health inequities. The South-East London Community Health study (SELCoH), the UK’s largest community epidemiological cohort study, highlighted the need to examine local data to get a clearer picture of health outcomes and inequalities particularly in urban communities, rather than relying on national figures, to better plan local services [28–30].

The prevalence of common mental disorders (CMD) in the SELCoH sample was 24.2%, and the study showed significantly poorer health outcomes in the Black Caribbean group compared to the Black African group, for almost all health indicators except hazardous alcohol use [28]. Phase 2 of SELCoH showed effects of discrimination on CMD was worse among recent migrants and Black and Mixed ethnic groups, with discrimination experiences generally most prevalent among the Black Carribean group [29]. SELCoH highlighted the importance of avoiding commonly made comparisons between wide ethnic groups, such as White versus Black [28]. Instead, SELCoH emphasised how intersecting identities of varying privilege and disadvantage (e.g., socioeconomic status, migration status, ethnicity etc.) show specific differences in odds for CMD, and highlight health inequalities [31]. A study of social networks and social support using SELCoH data showed differences across socio-demographic factors in types of social support and social networks [32]. The study found protective factors for CMD included perceptions of emotional and instrumental support, alongside the size of family and friend networks [32].

**Fig 1:**
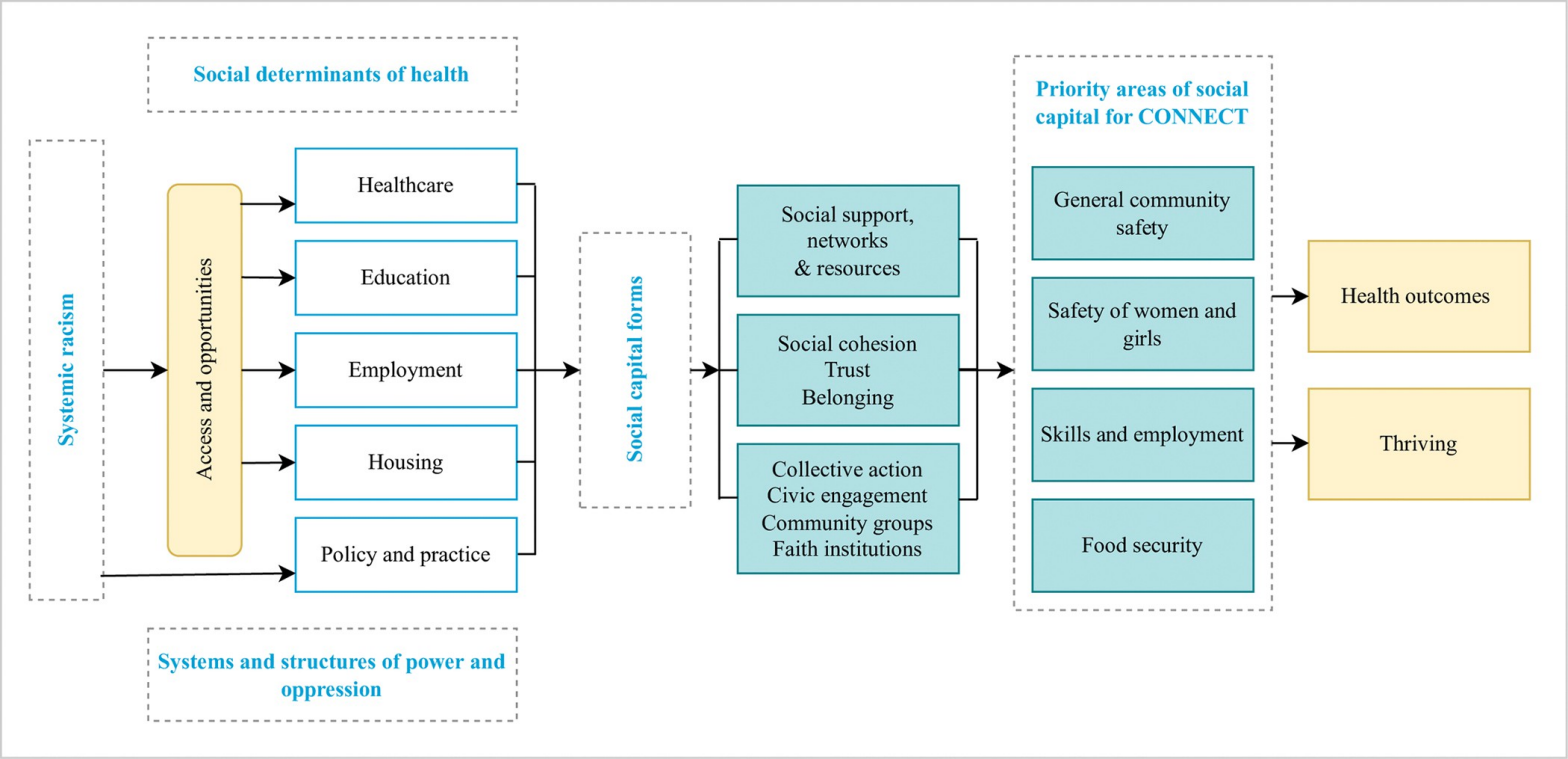
CONNECT theoretical model. This model illustrates that systemic racism affects access and opportunities, which can affect social capital and the four key areas our partners work in (promoting community safety, preventing violence against women and girls, promoting employment and skills and improving food security), and in turn impact on community thriving. This model has been adapted from Gilbert et al’s model [17] for Black social capital and social mobility in Black communities.

The CONNECT study builds on this previous research as well as on prior and ongoing trust and relationship building through the HERON network (https://heronnetwork.com), which was set up alongside SELCoH as a mechanism to support reciprocity and sustainable engagement. The study was designed within a partnership with community organisations and responds to local policy in two South-East London Boroughs, thereby providing potential channels for the action site of PAR. Collaborators for our study include leadership from local council, mental health charities and community organisations, who are key partners working in strategies to (1) promote community safety, (2) prevent violence against women and girls, (3) promote employment and skills and (4) improve food security. Fig 1 shows the CONNECT theoretical model which illustrates that systemic racism affects access and opportunities, which can affect the four key areas our partners work in, and in turn impact on community thriving.

Our study was driven by community engagement work carried out by above-mentioned partners who co-created the research proposal and protocol with the study team and identified specific areas that can inform their strategies and policies. For example, this research will be used to support improvements in the local authority’s children’s social care and services. As much of the domestic abuse seen in South-East London takes place within the context of children and families, the attention to prevent and build social capital should be focused here. As our study is closely aligned with our partners’ workstreams and strategies, this partnership will enable the voice of local people to be heard and will ensure the views and experiences of communities drive the changes they are committed to achieving.

### Study aims and objectives

The overall aim of this research is to investigate and understand how social networks and social capital act as resources that promote community thriving and identify ways to amplify social connections and improve mental health, focusing on the intersectional experiences of racially and ethnically minoritised communities. In our study, we will focus on four priority areas:

(a) community safety
(b) preventing violence against women and girls
(c) developing skills and employment
(d) food security

The objectives are:

1. To explore the role that social networks and social capital play in promoting thriving. We will investigate what the social networks are that people rely on in their day to day lives, what purpose these networks serve, including what resources (e.g., social, psychosocial, material, opportunistic) they offer, and what factors shape, constrain, and support them.
2. To understand how social relationships, networks, and their associated social capital generated by individuals and groups relate to mental health and place. We will map existing social capital embedded within social networks and how they are utilised at the community level (e.g., organisations, places), how individuals and groups utilise them, and how this relates to mental health.
3. To examine how both protective factors and adversities cluster, and how they are associated with cross-sectional and longitudinal trajectories of mental health using local and national quantitative data.

### A participatory action research approach

We believe communities know best what they need to thrive and be healthy [33,34], and that they should be involved in shaping how health and care support is provided [35]. However, researchers’ and even community organisations’ engagement with people from minoritised communities is often extractive and transactional, with resources being taken out of communities or organisations, rather than being put in [36]. Without reciprocity, a give and take approach, in which both parties are satisfied, power and resource imbalances are exacerbated, perpetuating inequality [37].

The CONNECT study will help overcome this gap by using a participatory approach [38] to knowledge generation and incorporating peer researchers from the local communities to optimise benefit for these communities and minimise power-based inequities in more traditional and university-led approaches to research [27,39]. PAR is based on a cycle of reflection, data collection, and action that aims to reduce health inequities through involving people from communities affected by the phenomenon under study who in turn take actions to improve health and wellbeing in their communities [40]. PAR emphasises (1) engagement with local perspectives and priorities [38]; (2) does research with/supports research by local communities (rather than doing research to, or about them); and (3) identifies ways to create change led by those most affected by the phenomenon being studied.

We will employ an anti-racism praxis to develop a successful community–institutional partnership, by acknowledging racism as a social construct and naming structural and institutional racism, increasing inclusivity and representation and strategising to dismantle power structures within our team [41,42]. We will do this by holding space for racially minoritised groups by including members of these communities as peer researchers in all stages of the study and engaging and financially recompensing organisations that are embedded or connected with populations we are working with.

### An integrated knowledge translation approach

To directly impact and lead to more equitable local policy and practice, our study will also adopt an Integrated Knowledge Translation (IKT) approach which is defined as “a model of collaborative research, where researchers work with knowledge users who identify a problem and have the authority to implement the research recommendations” [43]. IKT involves developing partnerships between researchers and “knowledge users”, or stakeholders, who utilise the research throughout the research process and apply the research into practice [44]. This means being inclusive not just by those who are affected by the research (e.g., peer or community researchers, community members, service users, health professionals), but also decision makers who have the power to enact the specific change researchers are hoping to achieve. These decision makers are included at all stages of the research process [45]. The relationships with knowledge users are created to enhance the relevance and uptake of research findings [9]. “Knowledge users” for our study include leadership from local council, mental health charities and community organisations mentioned above, who were involved with this study from its inception.

## Materials and methods

### Study design

**Fig 2:**
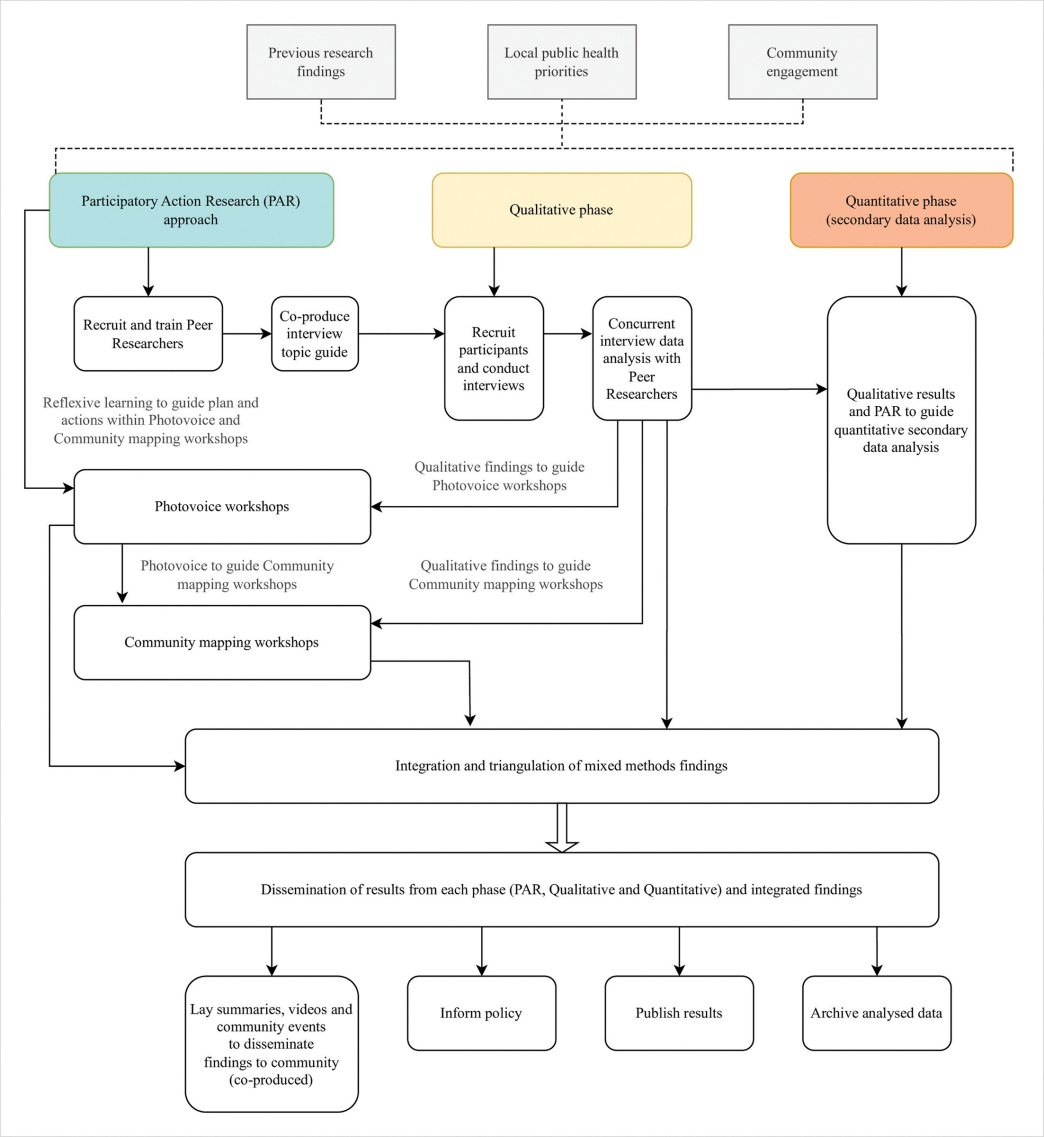
Flowchart of CONNECT study approach.

PAR research will be conducted using a sequential mixed methods approach involving both primary qualitative research and secondary quantitative data phases. Research will be carried out in multiple locations in the UK, however this protocol describes the procedure for the first geographical area only (two Boroughs in South-East London). We plan to expand the study to another urban area and an additional rural or coastal area in the UK, using our learnings from South-East London to shape how the study will be adapted and implemented in other locations. Fig 2 shows a flowchart of the approach and steps needed to complete the study.

### Implementation of PAR approach and collaborative research model

Our PAR approach [38] will involve recruiting up to ten peer researchers who (1) identify as being from Black or other racially minoritised groups, (2) are over the age of 16 years, and (3) reside in the study area. Selection criteria will also include (1) having a deep knowledge of the study area, (2) being active members of the community, (3) being passionate about health equity and supporting all members of the community, and (4) having some experience in, or knowledge of, community engagement, volunteering with local organisations or qualitative research methods. Peer researchers will be recruited through local community organisations and snowball sampling at the beginning of the project. All peer researchers will undergo appropriate training, through a free online research methods course (Research Methods: A practical guide to peer and community research) developed by members of our research team (CW and AE) and additional bespoke training sessions dedicated to conducting semi-structured interviews and community mapping workshops. This training will include 1:1 follow up and evaluation with experienced researchers from the wider research team to ensure competency. The research team will collectively engage in reflective practices on their own positionality in the research process. Finally, all members of the research team, including peer researchers, are required to undergo the same General Data Protection regulation (GDPR) and safeguarding policy training. In the event of peer researchers leaving the study team, they will be replaced using the same recruitment techniques. New peer researchers will have access to video recordings and detailed notes of all training sessions and will be supported through one to one guidance from an existing research team member.

A priority and moral responsibility of PAR is “a duty of care”, to focus on the safety and support of peer researchers throughout the research process. The online research methods course used for training includes a module about why it is important to stay safe and how to protect oneself as a researcher. There will be additional team discussion and reflection sessions around safety and support, and we will co-produce a safety and support protocol. Additional wellbeing support in the form of group debrief sessions, monthly one-to-one support and weekly optional drop-in sessions will be offered. Peer researchers will also have access to speak to a clinical psychologist who is part of the wider research team.

Through the training process, and following this, peer researchers will be involved in developing research questions, co-learning opportunities such as delivering training based around their own expertise, collecting primary qualitative data, transcribing interviews, supporting analysis of primary data, refining topic guides and contributing to research outputs.

Peer researchers will be paid at an hourly rate of £25, subject to any relevant tax and national insurance contributions, for up to 5 hours per week, for 12 months. We will also work with individual researchers to identify goals relevant to their personal development and identify appropriate opportunities to help them achieve their goals. This approach will help build community research capacity and enable researchers to participate as valued partners in the research process [46,47].

**Fig 3:**
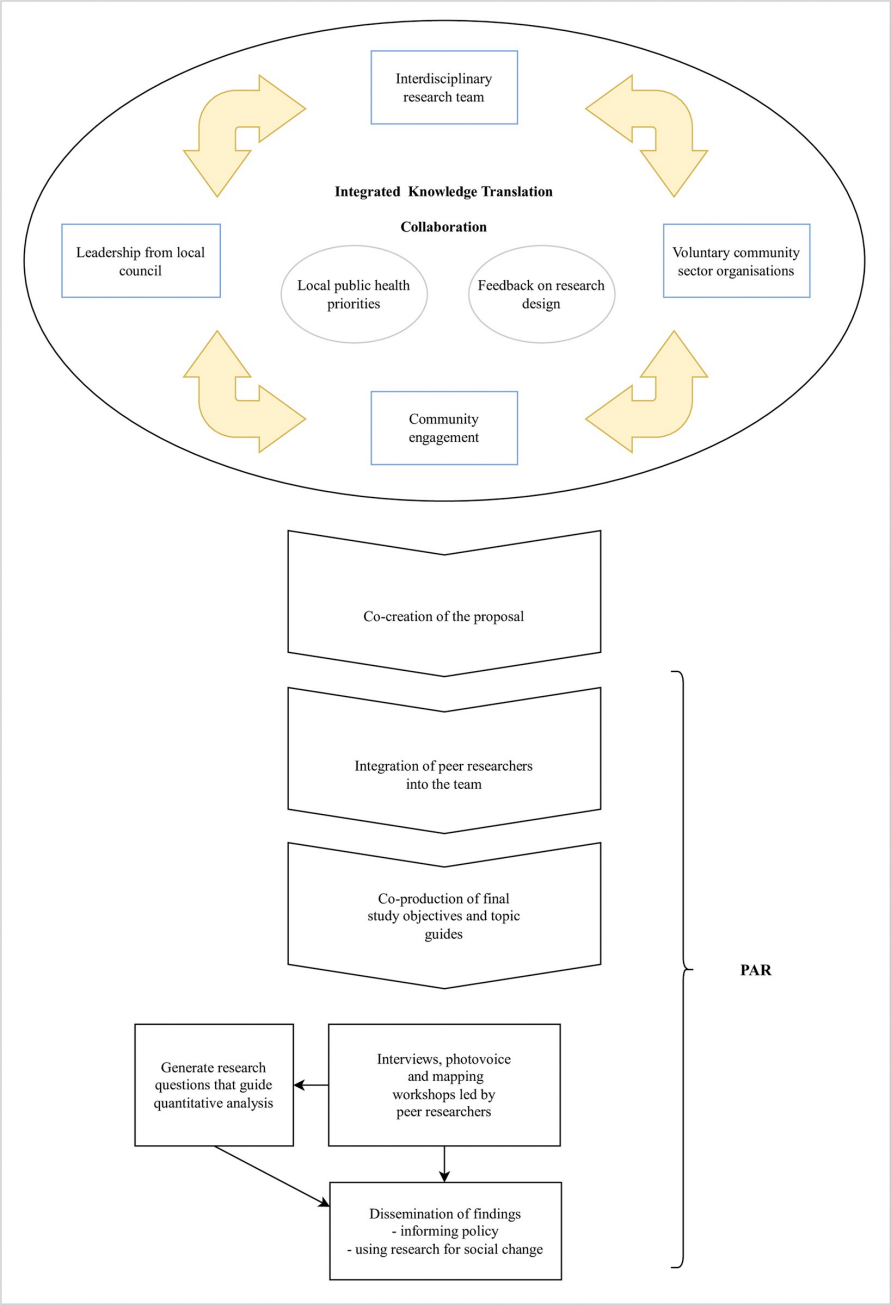
Collaboration and co-production model.

In addition to working with community members, we will take an IKT [43] approach, whereby the study team will collaborate closely with leadership from local council and local community organisations throughout. Close collaboration will be achieved through regular advisory and update meetings as well as written updates and feedback. The frequency of updates and meetings will vary from biweekly to quarterly with different partners and collaborators, guided by stakeholder mapping. Our model of collaborative research is outlined in Fig 3. The collaborators’ feedback was used to identify priority research questions for the project to address. Additionally, the collaborators were asked to provide feedback on draft interview questions, with particular attention given to the value of the information that would be gathered. Further improvements will be made to the interview questions based on collaborator’s responses and feedback throughout. Our research team, including peer researchers, will work collaboratively to create a framework that establishes a ‘way of working’ as part of the PAR and IKT process. This will ensure that roles and responsibilities are explicitly discussed and recorded. Every effort will be made to foster democratic, dynamic, and iterative processes in the co-creation of knowledge [48], however ultimate leadership of, and responsibility for, the project lies with the university researchers. This responsibility includes coordination of recruitment and data collection, analysis, and outputs, as well as planning and facilitating consistent meetings with collaborators to enable regular reflection and feedback throughout, as well as action on, and implementation of, findings. Data ownership will be shared with collaborating organisations as per existing data sharing agreements.

### Mixed methods approach

Research using mixed methods combines qualitative and quantitative approaches to integrate numerous perspectives, to amplify the strengths and reduce the limitations of each approach, and to gain a fuller understanding of the data [49]. Through a sequential mixed methods approach we aim to triangulate a deeper understanding of findings across qualitative and quantitative research phases, particularly knowledge generated through the lived experiences and expertise of peer researchers using PAR. The study’s sequential mixed methods approach is detailed in Fig 2.

The qualitative phase and quantitative phase of the mixed methods approach are described below. Findings stemming from the qualitative interview phase (objective 1) and reflexive learning through the PAR process will guide the quantitative analyses, and the focus of other sequential qualitative methods, such as Photovoice and community mapping workshops (objectives 2 and 3). For example, interview findings may guide which quantitative variables are potential mediators or moderators linked to social capital, in the associations between food security and mental health. Key results from each method and research phase, including PAR insights, will be triangulated for commonalities and inconsistencies between the results to produce thematic maps [50]. Meta-themes will be identified from cyclical discussions of findings and thematic maps with peer researchers and will be presented as integrated results. The cyclical process within PAR enables critical reflexivity, an opportunity to use findings through the process to tailor our study’s focus and analysis, e.g., within Photovoice, community mapping and quantitative analysis, and determine which actions ultimately become priorities. Incorporating a PAR approach across the mixed methods allows us to generate richer collaborative and integrated knowledge to inspire decision making, offer actionable insights, and challenge imbalances of power and resources across council strategies, local policies, and community enterprises.

### Qualitative phase (objectives 1 and 2)

#### Participants

The inclusion and exclusions criteria for study participants are listed in Table 1.

**Table 1:** Eligibility criteria.

	Number	Inclusion criteria	Exclusion criteria
**Semi-structured interviews**	Up to 70	• Aged 16 and over • Residing in study area within the past 3 years • Identifies as being Black or a member of another racially minoritised group	• Aged 15 or under • Does not reside in study area now or has not within the past 3 years • Does not identify as being Black or a member of another racially minoritized group
**Photovoice**	Up to 15 participants, 2 groups		
**Community Mapping Workshops**	Up to 20 participants per workshop, 3 workshops = 60 total		

#### Sampling

We will conduct up to 70 semi-structured interviews (to address objective 1). We are aiming for this sample size to ensure data are collected to allow for detailed analysis of the four key topic areas (community safety; preventing violence against women and girls; developing skills and employment; food security). This is important as we will use findings to inform recommendations and actionable insights for local council and community organisations. Saturation is planned to be achieved at the group level (i.e., saturation in each of the four key topic areas).

We will carry out two Photovoice workshops with up to 15 participants each to allow for trust building among participants, which will help guide the discussion and reflection following individual photo taking. Having up to 30 participants overall will allow for a range of new insights and perspectives of their communities and create a range of photographic recordings and evidence to raise awareness and motivate change.

We will host three community mapping workshops with up to 20 participants each. Having three groups will allow us to stimulate discussions through a variety of different types of community mapping (e.g., mental maps–how people perceive areas; activity maps–where people socialise; hazard maps–safe/risky places; resource maps–where people access different resources). Including up to 20 participants in each group will allow us to have a diverse group, to ensure representation of different characteristics and to give us an idea of the different views and perceptions of their neighbourhoods, as well as the resources that exist within their communities.

#### Recruitment

Recruitment will begin on 1^st^ June 2023. We will use a mix of opportunistic, snowball and purposeful maximum variation sampling to recruit participants from a range of racially minoritised backgrounds, ages and intersecting identities. The project will be publicised via our websites, e-newsletters, and social media channels. We will also recruit participants through our collaborators’ social media and e-newsletters, as well as by e-mails to stakeholders and community organisation contacts, through distributing flyers, and by attending events and visiting community spaces to engage with the community in person. In addition, we may contact individuals who have agreed and consented to be re-contacted via other related research projects.

People who are interested in taking part will be able to contact the peer researchers by e-mail, study phone, or direct social media message. Peer Researchers can refer to a co-developed (1) interview protocol and a (2) risk and safety protocol, throughout the interview data collection process. All participants will be provided with a participant information sheet which will include full details about the study, what is involved, how we will process and store data, confidentiality and anonymity, information about study/data withdrawal, any potential benefits, or risks of involvement. Participant information sheets will clearly state that participation is voluntary, and participants may withdraw from the project at any time. All participants must provide written consent prior to taking part in the research. We will allow a minimum of 48 hours to follow up participants to ask if they had any questions and resupply a link to a consent form if it has not yet been completed. Up to two further contact attempts (for a total of three attempts maximum) will be made to those indicating an interest to participate.

Participants will also be invited to take part in photovoice, which is a qualitative method used for community-based participatory research to document and reflect upon people’s reality through photography, to empower minoritised populations and to promote positive social change [51,52]. Such a visual and interactive research method enables individuals and communities to show aspects of their lives in more creative ways [53,54]. Photovoice can capture things we struggle to express verbally and can help engage community and decision makers after the research [51].

We will invite all participants who took part in semi-structured interviews who provided specific consent to take part in a workshop, and additional stakeholders from community organisations in relevant areas to take part in a participatory community mapping workshop to better define which resources exist and how they are utilised. Information about the mapping workshops will be contained in the original information sheet and provided to any new potential participants (community organisation representatives) who had not taken part in an interview.

#### Procedure

Depending on participants’ and researchers’ preferences with regards to accessibility, health, safety, or any other reasons, the interviews will take place online (via Microsoft Teams or Zoom), via telephone, at the University campus, or in person at an appropriate community location. The interviews can take place as walking interviews in communities to gain a sense of how social relationships are linked to different places within the community, and how individuals identify with groups and places in these communities.

We will ask questions related to the role that social networks and social capital play in promoting thriving. We will investigate which networks people rely on in their day to day lives, what purpose these networks serve, whether they are threatened, and if so, by what/whom.

Participants will be reimbursed for their time to take part in the study interviews and workshops with an e-gift voucher, reflecting the amount of time involved in line with our previous and on-going studies and University guidelines for vouchers. Semi-structured interviews will last up to 1 hour and be reimbursed at £15 voucher per interview.

The interviews will be complimented with several photovoice groups. These photovoice groups will involve (up to 15) community members and 3 facilitators and will be carried out in a workshop-style format. The participant photographers will undertake a group training session which will focus on both photographic skills and guidelines, as well as ethical issues of informed consent, privacy, confidentiality, and anonymity. The photovoice workshops will be developed using an existing toolkit (South-East London Photography—SELPh) and adapted to the projects’ research questions and research objectives. Some of the key components of the photovoice workshops, split across 6 weeks, will include: (1) Recruitment of community members and facilitators to the group. These can include community members that are already involved in the interviews and community mapping workshop components of the study; (2) delivering an initial workshop and introducing photovoice. This will allow the group members to become familiar with the participatory method and understand the aims and objectives. In addition, it is important to highlight the ethical considerations through different scenarios and discussions and establish ground rules with the group; (3) Providing group members with cameras so that they can take photographs based on the initial workshop and related discussions. The group members can then bring their photographs to a dedicated workshop session where they will have the opportunity to discuss their photographs with other members and share the stories and context behind the photos; (4) Organising a public engagement event in the form of a photography exhibition to engage community members and decision makers.

Building on preliminary work with our partners (e.g., existing engagement work) and the semi-structured interviews, the research team will host three participatory community mapping workshops to help develop a social and physical map of different communities, that illustrate which resources exist and how they are utilised (to address objective 2). We will invite 20 participants per workshop (60 participants in total). The workshops will be designed in co-production with peer researchers and key partners based on the ‘ways of working’ framework. Workshops will be co-facilitated by at least one newly trained peer researcher, one more experienced researcher, and one community partner. Community mapping workshops will last 2 hours and participants will be reimbursed at £30 per workshop session.

These workshops will help (1) triangulate interview data, (2) identify any gaps, (3) develop a basis to address these gaps and (4) address where additional support is needed to help communities thrive. We will also explore the potential role/utility and feasibility of using other ways to map community social networks, places and assets/resources, using digital apps and visualisation of existing local data.

#### Data analysis and management

The PAR and IKT approaches used in this study emphasise integrating community members and other relevant stakeholders at each stage of the research process, including data analysis. The analysis will be flexible and iterative to include different perspectives, with findings informing the action research cycle of the PAR process.

Semi-structured interviews will be audio-recorded and transcribed verbatim by a researcher or university-approved transcribing service. Community mapping workshop data will be recorded through a mixture of notetaking, audio-recording (e.g., of presentations, Q&As, any breakout group sessions), online collaboration software such as Miro, Jamboard, White board, and screen shots or images of artefacts created by participants (not of people’s faces). All primary data sources will be de-identified (retaining a pseudonym) before inputting into NVivo to support analysis using a thematic analysis approach. Authors GG, ZA and SC will have access to information that could identify individual participants during data collection, if these participants have consented to being contacted about further participation in the study. This will be saved in password protected files and deleted once data collection is complete.

Reflexive thematic analysis [55,56] will be used to analyse qualitative data sources. The whole research team, including peer researchers, will be involved in the analysis of interview data together with partner organisations. This will involve familiarisation, open/descriptive coding, coding framework development, deductive and inductive identification of thematic patterns within and across datasets using a constant comparative approach. Second raters will help check interpretations against the data. We will examine patterns and relationships between themes.

### Quantitative secondary data analysis phase (objective 3)

Insights gained from participatory workshops and semi-structured interviews will be used to generate research questions that guide our quantitative analysis phase. This will ensure that our examination of associations between indicators of social networks and mental health are appropriate and useful. We can achieve this by analysing the patterns in which financial and other insecurities cluster together across different aspects of social status and their associations with trajectories of mental health both cross-sectionally and over time.

To explore these associations, we will use secondary datasets, such as the South East London Community Health study (SELCoH [28], the Adult Psychiatric Morbidity Survey (APMS) [57], and Understanding Society [58]. These datasets have racially and ethnically diverse samples and include indicators of adversity (e.g., discrimination, poor mental health) and socio-demographic measures (migration status, income, geographic information). These datasets are anonymised and either openly accessible via the UK Data Archive (e.g., Understanding Society, Welfare Conditionality) or available via collaboration with the Health Inequalities Research Group (HIRG) at King’s College London (RR is lead for the HIRG data advisory and has access to APMS data covering the purposes of this work).

All secondary data are available in already anonymised form and will be analysed by appropriately trained members of the research team. Secondary quantitative data will be analysed using Stata, R and MPlus. Descriptive statistics and appropriate regression models will be used to examine relationships between social network related indicators and mental health, accounting for potential confounders. In taking an intersectional approach, latent class analysis will be used to identify groups defined by multiple advantaged or disadvantaged statuses (e.g., Black migrant women). Multilevel modelling will enable examination of between-area variation, where appropriate. Linear mixed effects models and related methods will be used to assess longitudinal associations between financial insecurities and adversity with social and mental health outcomes.

### Ethical considerations

This research was approved by the King’s College London Research Ethics Committee for Psychiatry, Nursing and Midwifery on 10^th^ November 2021; approval number: HR/DP– 21/22-26357. Written informed consent will be obtained electronically.

### Status and timeline of the study

At the time of writing, seven peer researchers have been recruited in total and have begun training in research methods. Recruitment of participants will begin on 1^st^ June 2023 and will continue for approximately 7 months. The rest of the timeline will be co-developed as a part of the participatory process.

Due to the iterative nature of the study, amendments are likely. Any changes will be made through discussions with members of the community and project stakeholders, who are drawn from a wide range of policy, research, community initiatives and service delivery. This iterative process has guided the project from the beginning and will continue to do so beyond dissemination.

## Discussion

Due to the innovative methods used, this study will provide an important foundation and precedent for the future development of community research. The processes developed for this study can be used as a tool in other research where the goal is to examine inequities in marginalised communities and engage the community throughout the research process. This is important as genuine social change requires a commitment to these types of research strategies that make apparent, continuously reflect upon, and minimise power imbalances within the research process.

Despite the strengths and novelty of the study design, several limitations should be highlighted. Although there are clear principles of PAR (community engagement, partnership, action, and change) there continue to be pitfalls in the implementation of this approach that further perpetuate structural and institutional racism [41]. Although PAR, in its most ethical use, engages community and academic partners in shared decision making, resource allocation, and power distribution, the application of this approach often falls short in addressing the inequitable distribution of power and resources among community—academic partnerships.

### Dissemination and implications for policy, research, and practice

The plan for dissemination of findings is substantial to the nature of this co-produced research. Sharing findings with local communities and those directly concerned by the research is our dissemination priority. Through co-production with peer researchers, we will ensure engagement and dissemination is designed in a way that promotes equitable access (i.e., in formats or forums that enable equitable participation and engagement) such as art-based knowledge translation, using various genres, such as visual arts, creative writing, and multimedia including video and photography (such as photovoice), to communicate research [59].

Dissemination will also consist of academic peer-reviewed journals, summary reports, and lay summaries, as well as presentations internally, and at conferences. Other outputs will be developed and shared with non-academic policy, professional, and public audiences, including local councils, service users, and community organisations. These will include community forums, panel discussions and podcasts. We will focus on the benefits the research provides for the community and will develop further outreach ideas with the peer researchers. Policy dissemination will be developed using stakeholder mapping exercises, capacity building workshops, engagement of policy makers in structured deliberative dialogues, and collaboration with public media [59]. Our IKT approach, with close partnerships with community organisations and local decision makers who have the authority to implement the research recommendations, will aid in ensuring the views and experiences of racially minoritised communities drive the changes we are collaboratively committed to achieving.

## References

[1] Brandow CL, Swarbrick M. Improving Black Mental Health: A Collective Call to Action. Psychiatr Serv. 2022;73:697–700. doi: 10.1176/appi.ps.202000894 34587786

[2] Milner A, Jumbe S. Using the right words to address racial disparities in COVID-19. Lancet Public Health. 2020;5:e419–20. doi: 10.1016/S2468-2667(20)30162-6 32707127 PMC7373398

[3] Sidanius J, Pratto F. Social Dominance: An Intergroup Theory of Social Hierarchy and Oppression [Internet]. 1st ed. Cambridge University Press; 1999 [cited 2023 Jun 23]. Available from: https://www.cambridge.org/core/product/identifier/9781139175043/type/book.

[4] Willen SS. Flourishing and health in critical perspective: An invitation to interdisciplinary dialogue. SSM—Ment Health. 2022;2:100045.

[5] Thrive London. Thrive Together Report.

[6] VanderWeele TJ, McNeely E, Koh HK. Reimagining Health—Flourishing. JAMA. 2019;321:1667. doi: 10.1001/jama.2019.3035 30933213

[7] Putnam RD. Bowling Alone: America’s Declining Social Capital. J Democr. 1995;6:65–78.

[8] Ehsan AM, De Silva MJ. Social capital and common mental disorder: a systematic review. J Epidemiol Community Health. 2015;69:1021–8. doi: 10.1136/jech-2015-205868 26179447

[9] Carpiano RM, Moore S. So What’s Next? Closing Thoughts for this Special Issue and Future Steps for Social Capital and Public Health. Soc Sci Med. 2020;257:113013. doi: 10.1016/j.socscimed.2020.113013 32418628

[10] Kindon S, Pain R, Kesby M, editors. Participatory Action Research Approaches and Methods [Internet]. 0 ed. Routledge; 2007 [cited 2023 Jun 23]. Available from: https://www.taylorfrancis.com/books/9781134135561.

[11] Feeney BC, Collins NL. A New Look at Social Support: A Theoretical Perspective on Thriving Through Relationships. Personal Soc Psychol Rev. 2015;19:113–47. 10.1177/1088868314544222 PMC5480897 25125368

[12] Holt-Lunstad J. Social Connection as a Public Health Issue: The Evidence and a Systemic Framework for Prioritizing the “Social” in Social Determinants of Health. Annu Rev Public Health. 2022;43:193–213. doi: 10.1146/annurev-publhealth-052020-110732 35021021

[13] Sun J, Harris K, Vazire S. Is well-being associated with the quantity and quality of social interactions? J Pers Soc Psychol. 2020;119:1478–96. doi: 10.1037/pspp0000272 31647273

[14] Machalek R, Martin MW. Sociobiology and Sociology: A New Synthesis. Int Encycl Soc Behav Sci [Internet]. Elsevier; 2015 [cited 2023 Jun 23]. p. 892–8. Available from: https://linkinghub.elsevier.com/retrieve/pii/B9780080970868320104.

[15] Bourdieu P. The Forms of Capital. Handb Theory Res Sociol Educ Ed J G Richardson N Y Greenwood Press. Greenwood Press; 1986. p. 241–58.

[16] Villalonga-Olives E, Kawachi I. The dark side of social capital: A systematic review of the negative health effects of social capital. Soc Sci Med. 2017;194:105–27. doi: 10.1016/j.socscimed.2017.10.020 29100136

[17] Gilbert KL, Ransome Y, Dean LT, DeCaille J, Kawachi I. Social Capital, Black Social Mobility, and Health Disparities. Annu Rev Public Health. 2022;43:173–91. doi: 10.1146/annurev-publhealth-052020-112623 34990220 PMC10195010

[18] Portes A. Social Capital: Its Origins and Applications in Modern Sociology. Annu Rev Sociol. 1998;24:1–24.

[19] Selvarajah S, Corona Maioli S, Deivanayagam TA, De Morais Sato P, Devakumar D, Kim S-S, et al. Racism, xenophobia, and discrimination: mapping pathways to health outcomes. The Lancet. 2022;400:2109–24. 10.1016/S0140-6736(22)02484-9 36502849

[20] Gazard B, Chui Z, Harber-Aschan L, MacCrimmon S, Bakolis I, Rimes K, et al. Barrier or stressor? The role of discrimination experiences in health service use. BMC Public Health. 2018;18:1354. doi: 10.1186/s12889-018-6267-y 30526564 PMC6286602

[21] Kapadia D, Zhang J, Salway S, Nazroo J, Booth A. Ethnic Inequalities in Healthcare: A Rapid Evidence Review. 2022;

[22] Williams DR, Lawrence JA, Davis BA. Racism and Health: Evidence and Needed Research. Annu Rev Public Health. 2019;40:105–25. doi: 10.1146/annurev-publhealth-040218-043750 30601726 PMC6532402

[23] Villalonga-Olives E, Almansa J, Knott CL, Ransome Y. Social capital and health status: longitudinal race and ethnicity differences in older adults from 2006 to 2014. Int J Public Health. 2020;65:291–302. doi: 10.1007/s00038-020-01341-2 32086535 PMC9951554

[24] Villalonga-Olives E, Wind TR, Kawachi I. Social capital interventions in public health: A systematic review. Soc Sci Med. 2018;212:203–18. doi: 10.1016/j.socscimed.2018.07.022 30048843

[25] Brune NE, Bossert T. Building social capital in post-conflict communities: Evidence from Nicaragua. Soc Sci Med. 2009;68:885–93. doi: 10.1016/j.socscimed.2008.12.024 19155112

[26] Benjamin GC, Jones CP, Davis Moss R. Editorial: Racism as a Public Health Crisis: From Declaration to Action. Front Public Health. 2022;10:893804. doi: 10.3389/fpubh.2022.893804 35747776 PMC9210344

[27] Campbell C. Social capital, social movements and global public health: Fighting for health-enabling contexts in marginalised settings. Soc Sci Med. 2020;257:112153. doi: 10.1016/j.socscimed.2019.02.004 30857750

[28] Hatch SL, Frissa S, Verdecchia M, Stewart R, Fear NT, Reichenberg A, et al. Identifying socio-demographic and socioeconomic determinants of health inequalities in a diverse London community: the South East London Community Health (SELCoH) study. BMC Public Health. 2011;11:861. doi: 10.1186/1471-2458-11-861 22078667 PMC3227613

[29] Hatch SL, Gazard B, Williams DR, Frissa S, Goodwin L, Hotopf M. Discrimination and common mental disorder among migrant and ethnic groups: findings from a South East London Community sample. Soc Psychiatry Psychiatr Epidemiol. 2016;51:689–701. doi: 10.1007/s00127-016-1191-x 26875153 PMC4846681

[30] Hatch SL, Woodhead C, Frissa S, Fear NT, Verdecchia M, Stewart R, et al. Importance of Thinking Locally for Mental Health: Data from Cross-Sectional Surveys Representing South East London and England. Zhang XY, editor. PLoS ONE. 2012;7:e48012. doi: 10.1371/journal.pone.0048012 23251330 PMC3520993

[31] Goodwin L, Gazard B, Aschan L, MacCrimmon S, Hotopf M, Hatch SL. Taking an intersectional approach to define latent classes of socioeconomic status, ethnicity and migration status for psychiatric epidemiological research. Epidemiol Psychiatr Sci. 2018;27:589–600. doi: 10.1017/S2045796017000142 28390448 PMC6998994

[32] Smyth N, Siriwardhana C, Hotopf M, Hatch SL. Social networks, social support and psychiatric symptoms: social determinants and associations within a multicultural community population. Soc Psychiatry Psychiatr Epidemiol. 2015;50:1111–20. doi: 10.1007/s00127-014-0943-8 25106666 PMC4464053

[33] Cahill C. Including excluded perspectives in participatory action research. Des Stud. 2007;28:325–40.

[34] Minkler M. Using participatory action research to build healthy communities. Public Health Rep. 2000;115:191–8. doi: 10.1093/phr/115.2.191 10968753 PMC1308710

[35] Public Health England. A guide to community-centred approaches for health and wellbeing. 2015.

[36] Chicago Beyond Equity Series. Why am I always being researched? [Internet]. 2019. Available from: https://chicagobeyond.org/researchequity/.

[37] Mezzina R, Gopikumar V, Jenkins J, Saraceno B, Sashidharan SP. Social Vulnerability and Mental Health Inequalities in the “Syndemic”: Call for Action. Front Psychiatry. 2022;13:894370. doi: 10.3389/fpsyt.2022.894370 35747101 PMC9210067

[38] Baum F. Participatory action research. J Epidemiol Community Health. 2006;60:854–7. doi: 10.1136/jech.2004.028662 16973531 PMC2566051

[39] Shiell A, Hawe P, Kavanagh S. Evidence suggests a need to rethink social capital and social capital interventions. Soc Sci Med. 2020;257:111930. doi: 10.1016/j.socscimed.2018.09.006 30219489

[40] Alwin DF, Felmlee DH, Kreager D, editors. Social networks and the life course: integrating the development of human lives and social relational networks. Cham, Switzerland: Springer; 2019.

[41] Adkins-Jackson PB, Vázquez E, Henry-Ala FK, Ison JM, Cheney A, Akingbulu J, et al. The Role of Anti-Racist Community-Partnered Praxis in Implementing Restorative Circles Within Marginalized Communities in Southern California During the COVID-19 Pandemic. Health Promot Pract. 2023;24:232–43. doi: 10.1177/15248399221132581 36419256 PMC9703012

[42] Jones CP. Toward the Science and Practice of Anti-Racism: Launching a National Campaign Against Racism. Ethn Dis. 2018;28:231. doi: 10.18865/ed.28.S1.231 30116091 PMC6092166

[43] Kothari A, McCutcheon C, Graham ID. Defining Integrated Knowledge Translation and Moving Forward: A Response to Recent Commentaries. Int J Health Policy Manag. 2017;6:299–300. doi: 10.15171/ijhpm.2017.15 28812820 PMC5417154

[44] Graham ID, Kothari A, McCutcheon C. Moving knowledge into action for more effective practice, programmes and policy: protocol for a research programme on integrated knowledge translation. Implement Sci. 2018;13:22. doi: 10.1186/s13012-017-0700-y 29394932 PMC5797415

[45] Gagliardi AR, Berta W, Kothari A, Boyko J, Urquhart R. Integrated knowledge translation (IKT) in health care: a scoping review. Implement Sci. 2015;11:38. 10.1186/s13012-016-0399-1 PMC4797171 26988000

[46] Collins SE, Clifasefi SL, Stanton J, The Leap Advisory Board, Straits KJE, Gil-Kashiwabara E, et al. Community-based participatory research (CBPR): Towards equitable involvement of community in psychology research. Am Psychol. 2018;73:884–98. doi: 10.1037/amp0000167 29355352 PMC6054913

[47] Collins C, Dolata J, Pike E, Sehgal A. Increasing research capacity in community organizations: Findings from the Community Research Scholars Initiative. Eval Program Plann. 2023;96:102189. doi: 10.1016/j.evalprogplan.2022.102189 36436308 PMC9801679

[48] Jull J, Giles A, Graham ID. Community-based participatory research and integrated knowledge translation: advancing the co-creation of knowledge. Implement Sci. 2017;12:150. doi: 10.1186/s13012-017-0696-3 29258551 PMC5735911

[49] Creswell J, Plano Clark V. Designing and Conducting Mixed Methods Research. Third Edition. Thousand Oaks, CA.

[50] McCrudden MT, Marchand G, Schutz PA. Joint displays for mixed methods research in psychology. Methods Psychol. 2021;5:100067.

[51] Wang C, Burris MA. Photovoice: Concept, Methodology, and Use for Participatory Needs Assessment. Health Educ Behav. 1997;24:369–87. doi: 10.1177/109019819702400309 9158980

[52] Budig K, Diez J, Conde P, Sastre M, Hernán M, Franco M. Photovoice and empowerment: evaluating the transformative potential of a participatory action research project. BMC Public Health. 2018;18:432. doi: 10.1186/s12889-018-5335-7 29609576 PMC5879794

[53] Humpage L, Fozdar F, Marlowe J, Hartley L. Photovoice and refugee research: The case for a ‘layers’ versus ‘labels’ approach to vulnerability. Res Ethics. 2019;15:1–16.

[54] Plunkett R, Leipert BD, Ray SL. Unspoken phenomena: using the photovoice method to enrich phenomenological inquiry. Nurs Inq. 2013;20:156–64. doi: 10.1111/j.1440-1800.2012.00594.x 22381071

[55] Braun V, Clarke V. Using thematic analysis in psychology. Qual Res Psychol. 2006;3:77–101.

[56] Byrne D. A worked example of Braun and Clarke’s approach to reflexive thematic analysis. Qual Quant. 2022;56:1391–412.

[57] McManus S. Mental Health and Wellbeing in England: Adult Psychiatric Morbidity Survey 2014: A Survey Carried out for NHS Digital by NatCen Social Research and the Department of Health Sciences, University of Leicester. 2016.

[58] Institute for Social and Economic Research. Understanding Society: Waves 1–12, 2009–2021 and Harmonised BHPS: Waves 1–18, 1991–2009, User Guide. 2022.

[59] Kwan BM, Brownson RC, Glasgow RE, Morrato EH, Luke DA. Designing for Dissemination and Sustainability to Promote Equitable Impacts on Health. Annu Rev Public Health. 2022;43:331–53. doi: 10.1146/annurev-publhealth-052220-112457 34982585 PMC9260852

